# Telemedicine in Heart Failure During COVID-19: A Step Into the Future

**DOI:** 10.3389/fcvm.2020.612818

**Published:** 2020-12-09

**Authors:** Gregorio Tersalvi, Dario Winterton, Giacomo Maria Cioffi, Simone Ghidini, Marco Roberto, Luigi Biasco, Giovanni Pedrazzini, Jeroen Dauw, Pietro Ameri, Marco Vicenzi

**Affiliations:** ^1^Division of Cardiology, Fondazione Cardiocentro Ticino, Lugano, Switzerland; ^2^Department of Internal Medicine, Hirslanden Klinik St. Anna, Lucerne, Switzerland; ^3^Department of Anesthesia and Intensive Care Medicine, ASST Monza, Monza, Italy; ^4^Department of Cardiology, Kantonsspital Luzern, Lucerne, Switzerland; ^5^Dyspnea Lab, Department of Clinical Sciences and Community Health, University of Milan, Milan, Italy; ^6^Division of Cardiology, Azienda Sanitaria Locale Torino 4, Ospedale di Ciriè, Ciriè, Italy; ^7^Department of Biomedical Sciences, University of Italian Switzerland, Lugano, Switzerland; ^8^Department of Cardiology, Ziekenhuis Oost-Limburg, Genk, Belgium; ^9^Doctoral School for Medicine and Life Sciences, Hasselt University, Diepenbeek, Belgium; ^10^Cardiovascular Diseases Unit, IRCCS Ospedale Policlinico San Martino, Genoa, Italy; ^11^Department of Internal Medicine, University of Genoa, Genoa, Italy; ^12^Cardiovascular Disease Unit, Fondazione IRCCS Ca' Granda Ospedale Maggiore Policlinico, Milan, Italy

**Keywords:** COVID-19, coronavirus, telemedicine, heart failure, remote monitoring, virtual visits, forward triage, telerehabilitation

## Abstract

During the Coronavirus Disease 2019 worldwide pandemic, patients with heart failure are a high-risk group with potential higher mortality if infected. Although lockdown represents a solution to prevent viral spreading, it endangers regular follow-up visits and precludes direct medical assessment in order to detect heart failure progression and optimize treatment. Furthermore, lifestyle changes during quarantine may trigger heart failure decompensations. During the pandemic, a paradoxical reduction of heart failure hospitalization rates was observed, supposedly caused by patient reluctance to visit emergency departments and hospitals. This may result in an increased patient mortality and/or in more complicated heart failure admissions in the future. In this scenario, different telemedicine strategies can be implemented to ensure continuity of care to patients with heart failure. Patients at home can be monitored through dedicated apps, telephone calls, or devices. Virtual visits and forward triage screen the patients with signs or symptoms of decompensated heart failure. In-hospital care may benefit from remote communication platforms. After discharge, patients may undergo remote follow-up or telerehabilitation to prevent early readmissions. This review provides a comprehensive appraisal of the many possible applications of telemedicine for patients with heart failure during Coronavirus disease 2019 and elucidates practical limitations and challenges regarding specific telemedicine modalities.

## Introduction

The Coronavirus Disease 2019 (COVID-19) pandemic has caused considerable morbidity and mortality worldwide. Epidemiological data from China indicate that patients with concomitant cardiovascular disease are more likely to develop life-threatening complications from severe acute respiratory syndrome coronavirus 2 (SARS-CoV-2) infection (1–7). The risk of complications may be even higher in patients with heart failure (HF) because they are older and have more comorbidities, but also due to the specific characteristics of this syndrome (8). Lockdown of social activities has allowed limiting the spreading of SARS-CoV-2, but it has also decreased medical contacts. For HF patients, this might have led to late recognition and treatment of episodes of decompensation and missed opportunities for optimization of medical and nonmedical therapy. In addition, lifestyle changes adopted during lockdown, such as dietary changes, increased alcohol consumption and decreased physical activity, may trigger HF decompensations (9, 10).

Telemedicine represents a useful tool to prevent negative direct and indirect consequences of SARS-CoV-2, and the present situation might be the right moment to implement a structured telemedicine program in clinical practice. Its main benefits include guiding the treatment of patients in primary care to minimize the risk of disease transmission during referral, continuing to provide optimal treatment to the patients with cardiovascular disease who are isolated at home or are discharged from the hospital to prevent clinical deterioration, monitoring early signs of new onset or worsening HF, and reducing unnecessary visits to the hospital to decrease the incidence of cluster infections (11).

In this review, we provide an overview of the many possible applications of telemedicine, its limitations and challenges, in patients with HF during COVID-19.

## Impact of COVID-19 on the Management of Heart Failure

Already in the first months of the COVID-19 pandemic, the impact of cardiovascular comorbidities on disease course became clear in observational studies, indicating that patients with previous cardiovascular disease had higher COVID-19 disease severity and mortality (2, 6, 7). In addition, myocardial injury in COVID-19 has been broadly described (6, 7, 12, 13), which might further impair myocardial function and worsen prognosis in patients with known HF.

Patients with chronic HF represent a vulnerable group during a pandemic of infectious respiratory disease. Previous studies have shown that they are at increased risk for adverse consequences of seasonal influenza (14) and other causes of pneumonia (15). Furthermore, acute infections may trigger HF exacerbations (16).

The social and environmental effects of lockdown must also be mentioned. A significant decline in hospitalization rates for acute HF during the COVID-19 pandemic, compared to before the pandemic and each of the preceding 3 years, was described, which might be the consequence of fear for infection leading to reluctance to seek medical attention when needed (17). Notably, hospitalized patients had more severe symptoms on admission, possibly suggesting that patients have waited longer before presenting to the hospital or less severe cases did not come to the hospital at all. Further, lifestyle changes during lockdown, such as dietary changes, increased alcohol consumption and decreased physical activity, may trigger HF decompensations (9, 10).

Although lockdown represents a solution to prevent viral spreading, it may complicate regular follow-up visits, therefore encumbering optimization of medical therapy and limiting detection of development of complications or disease progression that may require a change in management.

**Figure 1:**
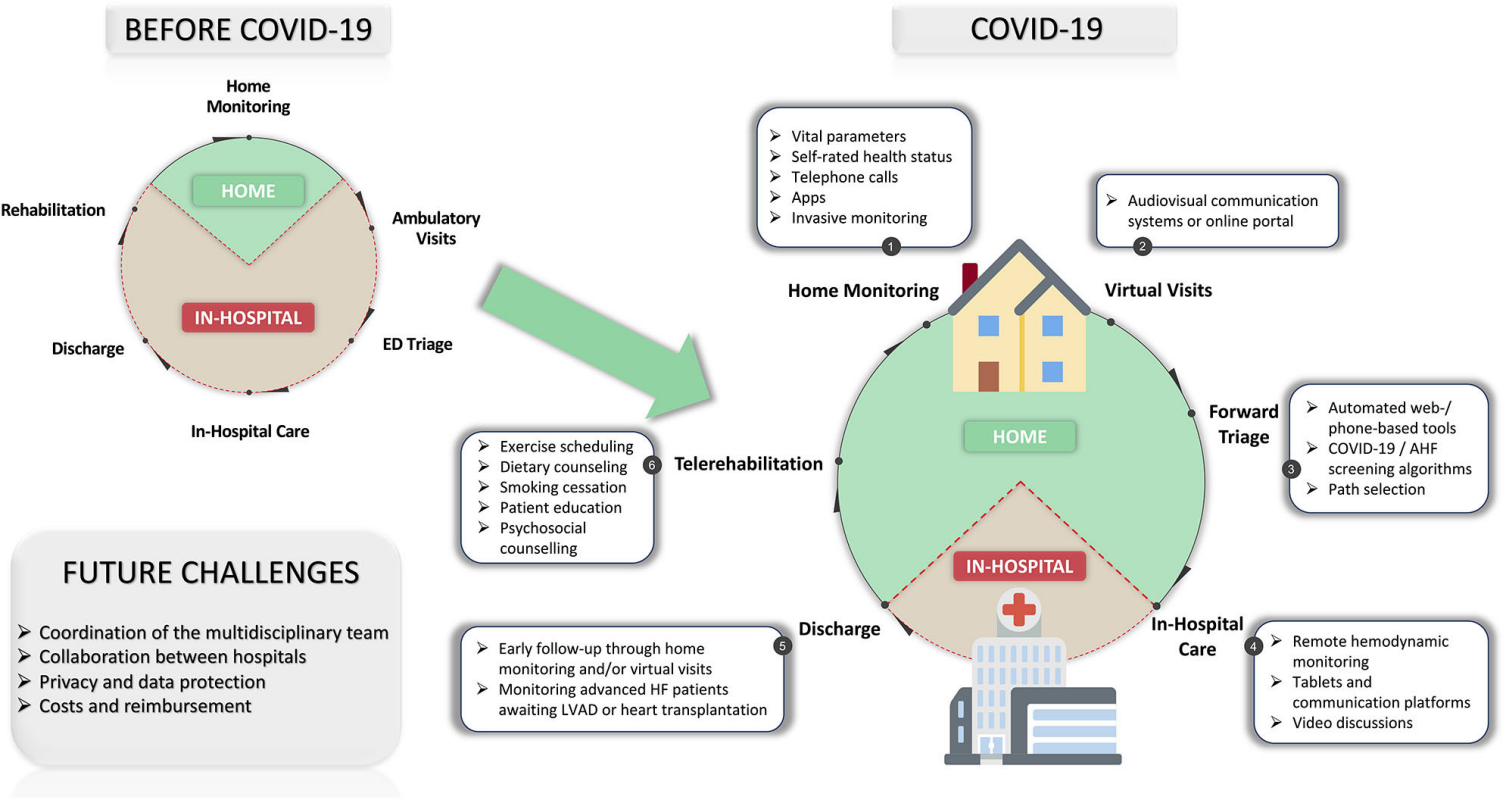
Telemedicine in patients with heart failure before and during COVID-19. AHF, acute heart failure; COVID-19, coronavirus disease 2019; ED, emergency department; LVAD, left ventricular assist device. Modified from https://github.com/emojione/emojione/tree/2.2.7 and https://github.com/twitter/twemoji/. Licensed under a CC BY-SA License (https://creativecommons.org/licenses/by-sa/4.0).

For these reasons, the great challenge of patients with HF during COVID-19 is keeping them safe from infection risk, but equally continuing with strict monitoring in order to prevent hospitalizations. As a result, health systems have largely transitioned to noncontact care delivery methods for ambulatory care (9). In this setting, various strategies of telemedicine and remote monitoring were developed rapidly and implemented more widely in HF patients (Table 1, Figure 1).

**Table 1:** Strengths and weaknesses of different telemedicine strategies for patients with heart failure during COVID-19.

**Strategies**	**Definition**	**Objectives**	**Challenges**
Home monitoring	Remote monitoring of vital parameters and transmission (via devices, telephone, apps) to a care center for interpretation and management	Individualized targets Therapy optimization Patients' empowerment Avoiding social disparities	Device delivery and patients' education Staff training Initial investment
Virtual visits	Remote visits with audiovisual telecommunication system or through an online portal	Assessment of symptoms Therapy optimization Maintain connection between patient and physician Seeing new HF patients	Adequate assessment of volume status or congestion Availability of stable internet connection and devices
Forward triage	Sorting of patients before presentation in the ED	Early assignation to the right path Protect patients from high-risk exposure	Logistic reorganization of ED triage models Software implementation
In-hospital telemedicine	Implementation of telemedicine in the in-hospital setting	Limiting unnecessary exposure to affected patients Favor communication and reduce social isolation	Staff training Hardware costs
Telerehabilitation	Delivery of rehabilitation services remotely	Allow cardiac rehabilitation during lockdown	Initial assessment Patients' compliance and motivation Costs and reimbursement

*ED, emergency department*.

## Telemedicine Strategies During COVID-19

### Home Monitoring

Several strategies can be applied to perform home monitoring of HF patients. Two small studies performed in Boston and New York City showed initial encouraging results of implantable hemodynamic monitoring in COVID-19 (18, 19). However, device and hemodynamic monitoring can only be performed in those patients, which had implanted a device or hemodynamic sensor before the lockdown, which are a minority of the HF population.

A new home monitoring system should be easy to install, be intuitive to users, and provide robust communication (20). Hence, structured telephone support (STS), defined as monitoring, self-care management, or both, delivered using telephone calls (21), may represent the most simple and affordable system for HF centers starting with telemedicine during COVID-19.

A recent study on 103 patients in an Italian tertiary referral center investigated whether a telemedicine service expressly set up during the COVID-19 outbreak changed HF outcomes compared with the same period of 2019 without telemedicine (22). Around 60% of patients accessed telemedicine services at least once, and half of contacts led to a clinical decision (e.g., adjustment of diuretic doses, change of blood pressure drugs, rate controls, and anticoagulant management). In this study, the telemedicine service reduced the composite of HF hospitalization and death compared to patients in the 2019 cohort, which is nevertheless to be interpreted cautiously in light of the previously mentioned reduction of HF hospitalizations during lockdown. In fact, new-established STS interventions are expected to give significant advantages only in the long term, since they could be influenced by a learning-to-care curve due to staff training (23). However, the main goal of telemonitoring during COVID-19 is not to provide superior care than standard, but to offer patients with HF a “health maintenance strategy” which provides an individualized target for each HF patient and adjusts treatment to maintain the monitored parameters as close as possible to ideal (20).

Besides HF patients in general, HF patients who suffer SARS-CoV-2 infection and are treated at home could even more benefit from STS as they are at high risk for complications (8). Remote monitoring can also encourage patients to maintain home isolation and assist in correct timing of stopping the isolation precautions (24).

### Virtual Visits

Virtual visits (VV) include remote visits, in which an audiovisual telecommunication system is used, and e-visits, which are communications between patients and providers through an online portal (9).

A recent statement from the Heart Failure Society of America provides information regarding platforms, workflows, and care models for VV in HF patients (25). Some institutions have already balanced the deferred or canceled face-to-face HF visits with rapid adoption of VV while employing several novel virtual health technologies with overall positive results (26). Specifically, the potential benefits of VV for HF patients are providing access to care and medical advice which would be otherwise difficult to obtain and reducing in-person exposure to SARS-CoV-2. Involvement of caregivers who may be present at home, but not in the outpatient clinic because of restrictions to hospital access, is an additional advantage of VV during the pandemic (25).

Hypothetically, this might represent also a smart working possibility for healthcare personnel, a class of workers for which this possibility is not usually considered or available.

VV may be best utilized for medication titration and optimization in stable patients with chronic HF. While substantial patient information can be gained from such visits, certain challenges remain, such as the adequate assessment of volume status or congestion (27). Thus, in-person visits should be reserved for recently hospitalized patients, patients approaching or with advanced HF, who are new post implantation of a left ventricular assist device (LVAD) or heart transplant, and those with new-onset HF (9).

### Forward Triage

Respiratory symptoms, as well as functional decline and fatigue, may be early signs of both COVID-19 and of decompensated HF. Hence, stratification of patients before arriving in the emergency department (ED), the so-called *forward triage*, represents another potential strategy for health care surge control.

Before COVID-19, many EDs modified their triage model by allowing a remote provider to perform intake (28). In an emergency situation, web-conferencing software with a direct line from a triage room to a clinician can be rapidly implemented (29). An automated web- or phone-based tool could guide HF patients with concerning symptoms to determine the need for self-isolation, symptom monitoring, urgent VV, or presenting to the ED (30). Through a structured telemedicine program, detailed medical and exposure histories might be easily obtained. Screening algorithms can be integrated and local epidemiological information can be used to standardize screening and practice patterns across providers (29). The ultimate goal is to guide patients to the right diagnostic–therapeutic pathway while protecting them from unnecessary risk and exposure.

Patients with suspected COVID-19 are isolated immediately upon arrival to emergency departments. In several centers in the USA, telemedicine carts (i.e., systems that integrate displays, cameras, microphones, speakers, and network access) were already successfully deployed into COVID-19 isolation rooms. This initiative increased provider/patient communication and attention to staff safety, improved palliative care and patient support services, lowered consumption of personal protective equipment, increased patient comfort, and reduced the psychological toll of isolation (31).

### In-Hospital Telemedicine

Certain principles of virtual medicine might be considered when approaching an HF patient seeking acute cardiac care during COVID-19. In this setting, telemedicine measures must aim at limiting unnecessary exposure to affected patients, utilizing remote hemodynamic monitoring and ICU flowcharts to evaluate patient progress and adjust medications (32). These data can be implemented with clinical assessments performed by a single bedside operator to generate operable conditions for safe, remote decision-making, using tools such as electronic stethoscopes and mobile ultrasound probes (32). Initial results of basic thoracic ultrasound programs in ICU are encouraging with rapid adoption of point-of-care ultrasound and commensurate reduction in formal imaging studies (26).

Importantly, COVID-19 has presented healthcare professionals with new and unusual barriers to effective communication between physician, patient, and family. As hospital visits are now frequently prohibited to patients' relatives, novel telecommunication and video options might be considered for patients to speak with loved ones, review treatment choices, and even discuss objectives of care (32). For this purpose, several hospitals introduced use of tablets and video calls with the ultimate goal to favor communication and reduce social isolation of hospitalized patients (33).

### Telerehabilitation

Cardiovascular rehabilitation (CR) represents a cornerstone in the treatment of patients with HF. The term *telerehabilitation* has been used in much of the literature to date and is defined as the delivery of rehabilitation services via information and communication technologies (34). Before COVID-19, it has been shown to be a viable and effective alternative for individuals who are unable to access in-person healthcare services for the management of many conditions. During COVID-19, the reallocation of medical resources as well as the lockdown caused the cessation of all nonurgent medical services, including CR. Therefore, centers had to switch to alternative ways to deliver the core components of CR remotely.

A technology-driven CR model has been proposed, with the assistance of any form of technology (e.g., smartphones, mobile apps, internet, e-mail, webcams, and use of wearable sensors) (35). A recent survey about the implementation of cardiac telerehabilitation services during the COVID-19 pandemic in Belgium (36) showed that half of the answering centers switched to telerehabilitation during the pandemic, mainly for patients that were already undergoing CR. The most frequently used medium to deliver the CR components were online videos (71%) followed by website information (64%) and emails (64%). As the authors of this survey suggested, the remote delivery of CR can also play an important role after the reopening of the rehabilitation centers because of a reduced capacity due to social distancing measures (36). For this purpose, a recent call for action paper of the European Association of Preventive Cardiology provides a practical guide for the setup of a comprehensive cardiac telerehabilitation intervention during the COVID-19 pandemic, which could also be relevant to any cardiovascular disease patient not able to visit CR centers regularly after the COVID-19 pandemic ceases (37).

### Advanced Heart Failure

The evaluation of patients with advanced HF awaiting LVAD placement or heart transplantation may be interrupted during the pandemic, as traditional social work, nutrition, pharmacy referrals, and diagnostic procedures are delayed. Telemedicine offers a platform for these multidisciplinary assessments to occur serially or simultaneously without delay (10). Furthermore, heart transplant recipients on stable immunosuppression at low risk for allograft rejection and hemodynamically optimized LVAD patients may be managed remotely without exposing them to further unnecessary risks (9). A telemonitoring algorithm for patients with LVAD has been recently proposed (38), and it is potentially adaptable to every LVAD center, regardless of the number of LVAD patients or previous experiences.

### Clinical Trials

Since the first wave of the pandemic, clinical trials unrelated to COVID-19 have been paused in most institutions. Telemedicine might avoid the loss of data during lockdown, which can jeopardize the entire research validity. In clinical trials, measurements and data collection are traditionally performed during patient visits. As stated by a recent document of the Heart Failure Association (39), endpoints like symptom status, quality of life questionnaires, or even vital signs could be assessed using home-based testing, with alternative methods such as telephone contacts, app-based self-assessments, or video links.

## Discussion

### Practical Considerations and Limitations of Telemedicine

Although telemedicine provides numerous advantages in many fields, it currently still carries practical limitations and pitfalls, which must be taken into consideration.

First, the hardware required for telemonitoring (i.e., smartphones, tablets, as well as blood pressure machines, scales, etc.) and exercise equipment for telerehabilitation (i.e., treadmill, stationary bike, etc.) may represent a significant financial burden, so either patients must be able to afford this or their health insurance/national health service must provide or reimburse the equipment. Moreover, patients who are unable to utilize the required devices or participate in a telemedicine session unaided either because of old age, poor hearing, cognitive dysfunction, language barriers, or limited education which may require the assistance of a family member or caregiver, who may not be available (40, 41). Finally, the use of telemedicine may be technically limited by poor phone and internet connectivity in rural areas (42, 43).

Telephone support is the most readily applicable and can be performed competently by trained nurses. However, home monitoring creates a large amount of data which must be screened and interpreted by trained staff (44), a process that could be time-consuming. In addition, it requires a dedicated physician to act on critical laboratory abnormalities, all of which can be challenging for physicians managing their practices and possibly receiving limited reimbursement.

The care of a patient with HF requires a multidisciplinary collaboration among physicians, pharmacologists, nurses, physical therapists, nutritionists, and medical social workers. Hence, technology should be conjugated also to ensure communication between the team (e.g., virtual multidisciplinary meetings using video calling in times of social restrictions) (37). In addition, patients with HF often have several comorbidities and may be looked after by more than one hospital, thus requiring intensive collaboration between different specialists and clinics. Authors analyzing the impact of the first COVID-19 wave on patients with chronic diseases described a poor interconnection between telemedicine services operating at higher levels (i.e., secondary or tertiary care facilities) and those deployed in primary care clinics or community pharmacies, preventing to obtain the maximum benefit from these digital solutions (45). Future developments should encourage the collaboration between different professional figures, departments, hospitals, and care institutions.

Due to the fact that telemedicine involves the transmission of patients' confidential information, whether those data are processed and transferred via telephone calls, videoconference, mobile apps, or other platforms, their monitoring requires safe encrypted storage systems which only allow for authorized access to data and protect patient privacy. The interfaces used must be compliant with local regulations both regarding data protection (i.e., GDPR) and encryption (i.e., HIPAA requirements) (46, 47). Physicians implementing telemedicine in clinical practice during COVID-19 suggest using device management software for telehealth devices to create security settings and enforce encryption for devices given to patients (48).

The inclusion of new patients in a telerehabilitation program will be challenging during lockdown, especially with respect to the initial assessment (i.e., baseline stress test) and initial interview, a hurdle that may be overcome by a structured technology-based program with predefined remote assessment methods and audio-visual communication systems (35). However, not all patients could be comfortable with this mode of action, and the problem of financing and delivering technologies to the single patients still persists. An effective approach to reorganize CR could be to start a rehabilitation path in person and subsequently integrate this with a patient-tailored remote telerehabilitation program in order to optimize performance and extend patients' education.

Finally, telemedicine services are not yet included in the essential levels of care in many countries (9, 29, 45). During COVID-19, some efforts were already made by agencies like the US Food and Drug Administration, which is facilitating the use of remote monitoring devices, and Centers for Medicare and Medicaid Services, which is paying for telehealth services at the same rate they would have been paid, if provided in person (27). However, these costs were covered only due to the emergency situation. In order to continue after the pandemic, the shift to telemedicine should be done in parallel with developments in policymaking (27).

### Future Perspectives

Evidence coming from observational studies on telemedicine during COVID-19 is of great importance. Centers having a dedicated HF unit should collect information regarding their own telemedicine approach, with the aim of defining strengths and weaknesses of each program and its impact on HF patients' care. This enormous amount of data provided during the pandemic should then be evaluated to be wisely implemented in daily clinical practice also after the crisis.

By evaluating results of telemedicine programs during COVID-19, one should keep in mind that in the particular setting of a pandemic, a system that is cost-efficient, user-friendly, and person-centered does not need to show that it improves outcome, but only that it is not inferior to traditional ways of delivering care and thus allows a safe maintenance of the *status quo* (20).

Although this pandemic has accelerated implementation of technology in the clinical setting, telemedicine should not be considered a cure-all for clinical scenarios. At its core, it remains a synergistic extension of the care team (49) and cannot entirely reproduce the bond-forming element of the traditional doctor–patient relationship based on direct face-to-face interactions (50).

## Conclusions

COVID-19 represents a serious threat for the HF population due to both higher risk of severe disease and death and reduced availability of outpatient care. Telemedicine in all its different forms and possibilities can be adopted to ensure continued healthcare delivery to patients with HF. Thus, we are witnessing its rapid, large-scale implementation during the pandemic. However, there are still several limitations and issues that should be solved in order to continue providing high-quality telemedicine services in patients with HF also after COVID-19.

## Author Contributions

GT and DW drafted the manuscript. GMC, SG, MR, LB, GP, JD, PA, and MV critically reviewed the manuscript. All authors have participated in the work and have reviewed and agreed with the content of the article. None of the article contents are under consideration for publication in any other journal or have been published in any journal.

## Conflict of Interest

The authors declare that the research was conducted in the absence of any commercial or financial relationships that could be construed as a potential conflict of interest.
